# Hepatitis B Virus-Related Cryoglobulinemic Vasculitis: Review of the Literature and Long-Term Follow-Up Analysis of 18 Patients Treated with Nucleos(t)ide Analogues from the Italian Study Group of Cryoglobulinemia (GISC)

**DOI:** 10.3390/v13061032

**Published:** 2021-05-30

**Authors:** Cesare Mazzaro, Luigino Dal Maso, Laura Gragnani, Marcella Visentini, Francesco Saccardo, Davide Filippini, Pietro Andreone, Anna Linda Zignego, Valter Gattei, Giuseppe Monti, Massimo Galli, Luca Quartuccio

**Affiliations:** 1 Clinical Experimental Onco-Hematology Unit, Centro di Riferimento Oncologico (CRO) IRCCS, 33081 Aviano, Italy; vgattei@cro.it; 2 Cancer Epidemiology Unit, Centro di Riferimento Oncologico (CRO) IRCCS, 33081 Aviano, Italy; dalmaso@cro.it; 3 MASVE Interdepartmental Center, Department of Experimental and Clinical Medicine, University of Florence, Center for Research and Innovation CRIA-MASVE, 50121 Firenze, Italy; laura.gragnani@unifi.it (L.G.); a.zignego@dmi.unifi.it (A.L.Z.); 4 Department of Translational and Precision Medicine, Sapienza University of Rome, 00185 Rome, Italy; marcella.visentini@uniroma1.it; 5 Rheumatology Unit, Internal Medicine Unit, Presidio Ospedaliero di Saronno, ASST della Valle Olona, 21047 Saronno, Italy; saccardo.merighi@aliceposta.it (F.S.); d.monti@tiscali.it (G.M.); 6 Rheumatology Unit, ASST Grande Ospedale Metropolitano Niguarda, 20162 Milan, Italy; davide.filippini@ospedaleniguarda.it; 7 Division of Internal Medicine, Department of Medical and Surgical Sciences, Maternal-Infantile and Adult, University of Modena and Reggio Emilia, 41124 Modena, Italy; pietro.andreone@unimore.it; 8 Infectious Diseases, L. Sacco Hospital, Department of Biochemical and Clinical Sciences, University of Milan, 20157 Milan, Italy; massimo.galli@unimi.it; 9 Rheumatology Clinic, Department of Medicine (DAME), ASUFC, University of Udine, 33100 Udine, Italy

**Keywords:** cryoglobulinemia, vasculitis, hepatitis B virus, entecavir, tenofovir

## Abstract

Hepatitis B virus (HBV) chronic infection causes progressive liver damage, although about 20% of patients develop extrahepatic manifestations such as cryoglobulinemic vasculitis (CV). Clinical manifestations range from mild to moderate (purpura, asthenia, arthralgia) to severe (leg ulcers, peripheral neuropathy, glomerulonephritis, non-Hodgkin lymphoma). A comprehensive review of therapeutic options for HBV-related CV is lacking. Nucleos(t)ide analogues (NA) suppress HBV replication in 90–100% of cases and induce clinical response in most patients with mild-to-moderate CV. Plasma exchange can be performed in patients with severe CV and should be considered in severe or life-threatening cases combined with high doses of corticosteroids and antiviral treatment. A cautious use of rituximab can be considered only in association with NA treatment in refractory cases. A review of the literature and an analysis of data collected by six centers of the Italian Group for the Study of Cryoglobulinemia on 18 HBV-CV nucleotide/nucleoside analogues (NAs)-treated patients were carried out.

## 1. Introduction 

Hepatitis B virus (HBV) infection is still a major global health problem with about 350 million chronically infected subjects worldwide. HBV infection can cause acute or fulminant hepatitis as well as chronic hepatitis evolving into cirrhosis and hepatocellular carcinoma, and it is responsible for 887,000 deaths every year [1]. About 20% of HBV patients may develop extrahepatic manifestations, such as polyarteritis nodosa and glomerulonephritis, dermatitis, arthralgia, arthritis, aplastic anemia and cryoglobulinemic vasculitis (CV) [2].

In the past, CV was termed “essential” due to its unknown etiology. After discovering hepatitis C virus (HCV) in 1989, it became clear that most CV cases were HCV positive [3,4]. 

CV can be described as an immune complex-mediated systemic vasculitis involving medium/small-size vessels. It is characterized by the presence, in the serum, of immunoglobulins able to precipitate when temperature goes below 37 °C [5]. According to Brouet and colleagues [6], cryoglobulinemias are classified into three types: I, II, and III [7]. In type I, the cryoglobulins are formed by monoclonal immunoglobulins, IgM or IgG only, and it is associated with lymphoproliferative disorders (multiple myeloma, Waldenstrom’s disease, or non-Hodgkin’s lymphoma, NHL). In types II and III, called mixed cryoglobulinemia (MC), the cryoglobulins are immunocomplexes composed by the antigen and monoclonal IgMs or polyclonal IgGs. The IgMs are usually endowed with rheumatoid factor (RF) activity against polyclonal IgGs. MC is strongly associated with HCV infection (80–90%) [8], but a fraction of cases is HCV-negative (10–20%), being secondary to other viral infections (HBV and HIV are the most common), or to systemic autoimmune diseases (primary Sjögren’s syndrome, systemic lupus erythematosus, and rheumatoid arthritis), or finally to chronic lymphoproliferative disorders [9,10,11,12,13,14,15,16,17,18,19,20]. MC can occur in 0.5 to 5.5% of HBV patients [21,22,23,24,25]. The potential role of HBV, as MC etiologic agent, was firstly suggested by Levo and colleagues [26] more than 40 years ago. Monti and colleagues [24] retrospectively analyzed a cohort of 717 subjects with essential cryoglobulinemia followed by the Italian Group for the Study of Cryoglobulinemia (GISC). HBsAg data were available only for 400 patients, and the authors reported a 5.5% prevalence of HBsAg positivity. Subsequently, Ferri and colleagues [23] evaluated 231 patients with MC, observing a 1.8% prevalence of HBsAg. In a recent study by Mazzaro and colleagues [27], the prevalence of HBsAg positivity in a group of 246 patients with MC was 4.5%. Furthermore, no correlation was found between MC and different HBV genotypes [2]. So far, no studies have evaluated the incidence of CV in HBV subjects. Our review focuses on clinical manifestations and treatments for HBV-related CV.

### 1.1. Main Clinical Manifestations of HBV-Associated CV

Since few clinical and epidemiological studies have suggested the casual relationship between HBV and MC (Table 1) [28,29,30], large population studies regarding HBV-related MC are lacking in the literature.

About 50% of HBV-MC patients show chronic hepatitis, while cirrhosis is present in 30% of cases.

The disease features vary: 45% to 100% of cases show mild–moderate clinical symptoms (palpable leg purpura, asthenia, and arthralgia, commonly called a Meltzer and Franklin triad [31]). The articular involvement is usually characterized by bilateral and symmetric joint pain, non-deforming, and mainly involve knees and hands. Skin ulcers may occur in 10–30% of cases. Sicca syndrome and Raynaud’s phenomenon have been reported in a few patients. Neurologic manifestations range from distal sensory polyneuropathy to sensory–motor polyneuropathy in 20–60% of cases. Peripheral neuropathy presents with leg pain and symmetric burning paresthesia. Motor deficit is irregular and mainly affects the lower limbs, appearing either a few months after sensory symptoms or simultaneously. Severe clinical symptoms such as glomerulonephritis, progressive peripheral neuropathy, gastrointestinal vasculitis, and NHL may occur in a few cases [22,28,29,30]. 

Similar to HCV-related CV, the most frequent kidney manifestation is type I membrano-proliferative glomerulonephritis (MPGN). A very common aspect of HBV-MPGN is nephrotic-range proteinuria and microscopic hematuria, often with evidence of renal insufficiency. In a recent study on 12 patients affected by HBV-MPGN [30], proteinuria was present with a nephrotic range in all of them, and 9 (75%) patients had impaired renal function. Microscopic hematuria was found in all patients, and gross hematuria in three. 

The histological picture found in MPGN has revealed diffuse endocapillary proliferation, thickening, and double-contour appearance of the glomerular basement membrane. The glomeruli were infiltrated by many monocytes and polymorph nuclear cells. The capillary lumen showed PAS-positive hyaline thrombi. The distinctive histological features are markedly hypercellular and endoluminal thrombi due to the massive precipitation of cryoglobulins. Immune complexes comprising HBV antigens were also detected in some cases [30]. Overall, kidney involvement emerged as an unfavorable prognostic factor [32,33]. 

### 1.2. Therapeutic Management of HBV-Related CV

HBV-associated CV is considered a rare disease and, consequently, few data are available regarding the clinical management, because large cohort studies are lacking. Furthermore, the implementation of universal HBV vaccination programs is successfully decreasing HBV infection prevalence worldwide [34], thus making HBV-associated CV progressively less frequent. 

Guidelines for treatment of HBV-related CV have not been published yet, but, similarly to HCV-related CV, the treatment is based on the following four targeting approaches: (1) antiviral therapy; (2) B-cell depleting therapy; (3) immunosuppressive drugs; and (4) anti-inflammatory drugs.

## 2. Antiviral Therapy

### 2.1. Oral Nucleot(s)ide Analogues (NAs)

Eradication or strong and effective suppression of HBV chronic infection by NAs is the first-line treatment for HBV-related CV. Table 2 summarizes the main studies on the treatment of HBV-related CV with NAs.

Some case reports have shown that viral suppression induced by NAs was associated with serum clearance of cryoglobulins, rheumatoid factor (RF) normalization and disappearance of purpura, arthralgia, Raynaud phenomenon and peripheral neuropathy [35,36,37,38,39,40,41,42]. In particular, Visentini and colleagues [43] reported the regression of VH1-69+ B-cell clones, which have been implicated also in the pathogenesis of HCV-related CV, in two patients successfully treated with antiviral therapy. In a wider population where NAs treatment was effective in suppressing HBV replication, a significant improvement of peripheral neuropathy was reported in 2/6 patients, while skin ulcers persisted in two [28]. However, other authors obtained skin ulcer improvement combining entecavir therapy with corticosteroids and plasma exchange [40]. In 2016, the GISC conducted a retrospective observational multicenter study, recruiting 17 HBV-DNA-positive CV subjects. Seven patients were treated with NAs for 48 months: five with entecavir, one with adefovir, and one with lamivudine [29]. After 12 months of NAs therapy, HBV-DNA was undetectable in all subjects, while HBsAg remained positive in all cases (100%). Purpura disappeared in all cases (100%), an improvement of arthralgia was observed in five patients (71%), while one patient experienced regression of leg ulcer. NAs induced a reduction of cryocrit levels in all cases, but only two patients showed undetectable levels of cryoglobulins during treatment. RF levels decreased in all seven patients, though a normal RF was observed only in one. C4 serum level remained low during treatment at 48 months of therapy.

Viganò and colleagues [40] reported purpura disappearance, reduction of cryoglobulins and normalization of RF, C4, creatinine and proteinuria in one patient treated with entecavir after negativization of HBV-DNA. Terrier and colleagues [22] studied 324 cases of non-HCV-related CV, including eight patients with HBV disease. Three patients with glomerulonephritis received lamivudine or entecavir with improvement of renal function and remission of purpura in one case while rituximab combined with entecavir was necessary to induce remission of nephropathy in the remaining two cases. 

In a recent study, Li and coworkers [30] described 12 patients affected by HBV-associated glomerulonephritis. Seven were treated with entecavir for 16 months and 2 with lamivudine for 12 months. The main clinical, biochemical, histological characteristics of the patients and NAs therapy are shown in Table 2. Nephrotic syndrome was present in nine subjects and a rapidly progressive glomerulonephritis in three. Nine patients had impaired renal function with a mean eGFR of 35.7 ± 24.3 mL/min at diagnosis. At the end of the follow-up (6–60 months), viremia was undetectable in all patients. In five patients treated with entecavir alone, a complete response was observed in two and a partial response in three; of these five cases, two had also purpura and arthralgia and one gastrointestinal vasculitis. Four patients were treated with steroids and immunosuppressants associated with NAs, but they had no response showing a progressive worsening of the renal failure requiring dialysis, and two of them died from sepsis, and cerebral hemorrhage, respectively. 

### 2.2. Pegylated-Interferon Alfa 

Compared with NAs therapy, Pegylated-interferon-α (PEG-IFN-α) has the advantage of finite treatment duration and slightly higher rates of HBsAg and HBeAg seroconversion. However, there are several contraindications and severe side effects, which discourage its use in HBV-related CV [44]. Löhr and colleagues [45] reported a single case of a young female with chronic hepatitis B and purpura due to type II MC treated with PEG-IFN-α 2b, three times weekly for twelve months. After one month of treatment, ALT returned to the normal range, while cryoglobulins disappeared from the serum. HBV-DNA was undetectable after two months, and purpura disappeared. Among the 17 patients with HBV-associated CV studied by Mazzaro and colleagues [29], PEG-IFN-α 5MU/day for six months was administrated to two patients with CV glomerulonephritis. In both, purpura and arthralgia did not improve and serum HBV-DNA remained positive. Also, the levels of cryocrit, RF, C4, creatinine and proteinuria remained unchanged and they were considered as non-responders. Visentini and colleagues [42] reported one case with HBV-related MC treated with PEG-IFN-α 2b. After six months of therapy, HBV-DNA was undetectable, purpura was resolved and cryocrit decreased, whereas C4 and RF remained stable. In addition, La Civita and colleagues [46] described a single HBV-positive MC patient with recurrent purpura, mild sensory peripheral neuropathy, and active hepatitis treated with PEG-IFN-α. The authors noted a rapid improvement of the purpura associated with ALT normalization and disappearance of cryocrit and serum HBV-DNA after four months of treatment. However, the concomitant worsening of the peripheral neuropathy prompted discontinuance of PEG-IFN-α. 

In summary, effectiveness of PEG-IFN-α in HBV-related CV is inconsistent. Right now, the NAs therapy should be preferred over PEG-IFN-α as first-line therapy in HBV-related CV.

### 2.3. Rituximab

Rituximab is a chimeric monoclonal antibody anti-CD20 antigen, selectively expressed in B-cells, which are expanded and abnormally activated in MC. Many reports have indicated that rituximab is effective in severe clinical manifestations of HCV-related MC, including skin ulcers, glomerulonephritis, peripheral neuropathy, and hyper-viscosity syndrome [47,48,49,50,51]. Pasquet and colleagues [52] reported a single case of HBV-associated type I membrano-proliferative glomerulonephritis, initially treated with corticosteroids in association with antiviral therapy (lamivudine). Because of a gradual worsening of renal function despite treatment with plasmapheresis, the patient received rituximab (375 mg/m^2^ in 4 weekly infusions) and lamivudine was substituted with entecavir. The purpura remitted, creatinine returned to normal, proteinuria, cryocrit, and RF decreased, and HBV-DNA became undetectable. Terrier and colleagues [22] used NAs therapy in 3 patients with glomerulonephritis obtaining improvement of renal function and remission of purpura in one. The other two patients received rituximab in combination with entecavir, leading to remission of kidney disease. Nonetheless, subjects treated with rituximab should be carefully monitored for infection complications. 

In summary, there are plenty of works [47,48,49,50,51] that demonstrate the effectiveness of rituximab for non-responders to the antiviral therapy, relapsing patients or for patients with severe or life-threatening CV (glomerulonephritis, peripheral neuropathy, extended cutaneous ulcers, gastrointestinal vasculitis, acute hyper-viscosity syndrome). A highly active antiviral therapy must always be associated with rituximab, not disregarding the risk of a possible viral reactivation in HBV-positive subjects, including those with a past infection (HBcAb-positive only) [44]. In fact, several investigators have reported fulminant hepatitis caused by HBV reactivation [53] after rituximab therapy. Moreover, HBV reactivation was reported even several months after the completion of rituximab treatment [5].

### 2.4. Plasma exchange

Removing circulating cryoglobulins by means of apheresis procedures is still considered the most efficient way to improve acute conditions in patients with severe CV [54]. Data from small series of patients and one small randomized study have shown that plasma exchange associated with immunosuppressive agents or corticosteroids was more effective in a severe CV than with plasma exchange alone [55,56,57]. A recent retrospective cohort survey including 22 Italian GISC centers evaluated 159 patients with severe CV who underwent apheresis [58]. 

The authors found that patients with life-threatening CV were significantly less likely to respond and significantly more likely to die during the first year after the initiation of the apheresis sessions (59%) than those without life-threatening CV; apheresis failure was associated with an increased risk of death. Cyclophosphamide has been used in association with apheresis to reduce the post apheresis rebound in cryoglobulinemic production [59], but it was only used in 19.5% of the patients reported by Marson and colleagues [58], mainly in association with the first apheresis session. The cost–benefit ratio of adding cyclophosphamide to apheresis is a subject of debate, and the choice should be evaluated in each case [60]. Data from small case series support the effectiveness of pulse high-dose corticosteroid therapy in controlling CV flares [59], and corticosteroids were associated with apheresis at different times and doses in treating 86% of the patients included in the study by Marson and colleagues [58]. In most reported cases, pulsed high-dose corticosteroid therapy was administered in association with the first apheresis session [58]. The guidelines of the American Society for Apheresis (ASFA) [61] include severe/symptomatic cryoglobulinemia amongst the disorders for which this procedure should be considered as second-line treatment. Few case reports have been published on plasmapheresis in HBV-associated CV. Terrier and colleagues [22] administered lamivudine, corticosteroids, and plasmapheresis to one patient with HBV-CV with nephropathy and purpura not obtaining a clinical response. Subsequently, the patient underwent rituximab, attaining clinical remission of purpura and nephropathy. A second patient affected by HBV-related nephropathy and purpura received entecavir, plasmapheresis, corticosteroid, and cyclophosphamide, obtaining clinical remission of nephropathy and purpura. In one recent study [30], two patients with HBV-related CV with rapidly progressive glomerulonephritis were initially treated with entecavir, corticosteroid, and plasmapheresis, without appreciable changes in the clinical and biochemical features. Subsequently, one of them was treated with rituximab, obtaining purpura remission, reduction of cryoglobulins and RF, but renal failure worsened, requiring dialysis. 

Globally, plasmapheresis may be indicated in severe and life-threatening HBV-related CV. However, in patients with HBV-positive cirrhosis due to either hemodynamic changes and the immunological dysfunctions associated with this condition, plasmapheresis should be used very cautiously.

### 2.5. Steroids

In high doses or as pulse therapy, corticosteroids have been in use for many years in subjects with severe systemic CV (ulcer, progressive peripheral neuropathy, and glomerulonephritis) [62]. The high doses of corticosteroids, both employed alone or associated with apheresis, are recommended by GISC to control severe CV flare [60]. 

A previous study [29] described the use of low-dose corticosteroids for 48 months in five patients, obtaining the remission of purpura and arthralgia in three patients while clinical symptoms persisted after the end of treatment in the remaining two. All patients obtained a reduction of the cryocrit level, but none of them showed a complete disappearance of cryoglobulins at the end of therapy. The RF serum levels remained elevated, and the C4 level remained unchanged at the end of treatment in all patients. Notably, in 8 patients treated with corticosteroids alone without NAs, no virological response was documented (HBsAg remained positive in all, with detectable HBV-DNA) [29]. 

Low-dose and long-term treatments with steroids should not be used for chronic viral infections and are responsible for significant side effects. However, as stated in the GISC recommendation, such a treatment may be helpful if administered as short-term courses for chronic pain control [63]. 

### 2.6. HBV-Related CV Treated with NAs: A Long-Term Follow-Up Analysis from GISC 

An update of previous results with a long follow-up analysis of HBV-CV patients treated with NAs is herein provided.

Six Italian centers, belonging to the GISC, were invited to provide retrospectively anonymous data in subjects who had undergone NAs therapy between June 2010 and February 2020, with a standardized aggregate data collection form. The mean follow-up was 75 months (range: 9–123). The only inclusion criterion for this study was the presence of CV symptoms in HBsAg positive patients. All cases met the eligibility criteria for treatment with NAs according to guidelines for the management of chronic HBV-infection [47].

The study comprised 18 patients with HBV-related CV, among whom seven had already been described in a previous paper [29], and they were included herein since they were still on NAs therapy and regularly monitored. Table 3 summarizes the main clinical, immune, and virological characteristics of the study population. 

Purpura was present in 18/18 (100%) patients, arthralgia in 11/18 (61%), ulcers in 3/18 (17%), Sjögren’s syndrome in 5/18 (28%), peripheral neuropathy in 11/18 (61%), glomerulonephritis in 1/18 (6%) and 2/18 (11%) had a NHL. Before the beginning of NAs therapy, 3 (17%) patients underwent treatment with PEG-IFN-α for 12 months and 2 patients achieved a transient vasculitis response despite a persistent HBV-DNA positivity. 

Four out of 18 (22%) patients received a low dose of prednisone (≤10 mg/day) associated with NAs therapy to control purpura flares and arthralgia. Plasma exchange associated with NAs was used in 4 (22%) patients with severe CV: One had elevated cryocrit in low-grade NHL, 1 had nephropathy, 2 had debilitating neuropathy and skin ulcers. Low doses of rituximab (250 mg/m^2^/weekly for four times) associated with NAs was used in 2 patients (11%): one had low-grade NHL and peripheral neuropathy, while the other had severe peripheral neuropathy and skin ulcers. Eleven patients were treated with entecavir for a median of 78 months (range: 9–111), 6 cases with tenofovir for a median of 67 months (range: 48–120), and 1 case with lamivudine for 58 months. After 6–12 months of therapy with NAs, viremia was undetectable in all patients (100%) and remained undetectable during the entire follow-up. In all patients, HBsAg remained positive. During the NAs therapy, purpura disappeared in 14/18 (78%), in 8/11 (73%) improvement of arthralgia, while regression of the leg ulcers in 2/3 (67%). Disappearance of leg ulcers was observed in one patient treated with entecavir monotherapy, while another patient treated with entecavir required plasma exchange followed by rituximab. A third patient showed skin ulcers persistence. Peripheral neuropathy improved in 5/18 (45%) cases (2 treated with entecavir and 3 with tenofovir). One patient with glomerulonephritis showed no improvement of renal function with tenofovir treatment and underwent plasma exchange and, subsequently, low doses of rituximab infusions. Despite the therapeutic efforts, the kidney failure progressed, requiring dialysis. A low-grade NHL case treated with tenofovir did not show a hematologic response and underwent sequential treatment with plasma exchange and low-dose rituximab, with a partial response. One patient on entecavir developed a cerebral diffuse large B-cell lymphoma after 60 months of therapy. Despite chemotherapy, the patient died because of lymphoma progression. One patient with cirrhosis and CV was treated with entecavir and obtained a rapid improvement of the purpura, while the peripheral sensory neuropathy persisted. Despite virological suppression and reduction of cryocrit and RF, the patient died of decompensated cirrhosis after 60 months. NAs therapy induced a decrease in cryocrit levels in all cases, although only 6/18 (33%) showed disappearance of cryoglobulins. The RF decreased in all patients, but the C4 serum levels remained low during treatment. No exacerbations of the clinical manifestations and no side effects were observed. 

Long-term therapy with NAs should take into account other factors such as the patient’s treatment compliance, the possible development of viral mutations causing drug resistance, and the potential toxicity, although the availability of different NAs guarantees a therapeutic coverage with multiple options. Treatment with NAs in subjects with HBV-related CV should be continued indefinitely even after the CV symptoms disappeared. The NAs treatment can be stopped only for those patients who achieve complete recovery from CV, HBsAg loss and HBsAg seroconversion.

Based on these reports, we can state that an optimal treatment for HBV-associated glomerulonephritis has not yet been established. The therapeutic schedule always includes the antivirals, but when HBV-CV is associated with nephrotic syndrome and a rapid decrease of renal function, the use of rituximab and plasma exchange should be considered [47,48,49,50,51,52].

## 3. Discussion

CV occurs in a smaller fraction of HBV patients, and for this reason only case reports or studies on a limited number of subjects are available in the literature. Nevertheless, the clinical manifestations are similar to those induced by HCV, varying from mild–moderate symptoms to severe and life-threatening manifestations (Table 4). CV pathogenesis in HBV patients remains largely unresolved even if it has been suggested that HBV antigens (as well as HCV) stimulate, through CD81, the B-cell clones producing IgM-RF [62]. Therefore, HBV-related CV could represent an evolution from polyclonal B-cell population to oligoclonal B-cell expansion in the setting of a long-term antigen stimulation, and it is considered a transitional phase between autoimmunity and neoplasm [64,65].

No specific treatment guidelines are currently available for patients with HBV-related CV and the best treatment has yet to be established. As demonstrated in HCV-related CV, direct antiviral agents are effective in a significant proportion of patients and usually introduced as the first-line treatment [66]. The literature reviews herein described, together with the original results we reported in a long-term follow-up of the largest series of patients treated with NAs, suggest that HBV replication is the triggering factor, and antiviral therapy may be the first-line treatment. The suppression of HBV replication is the goal to obtain CV remission, to prevent organ complications, and the possible evolution to lymphoproliferative disorders [67], which is a common neoplastic evolution in cryoglobulinemias, with possible underlying similar hyperactivated pathways and risk factors [68]. 

## 4. Conclusions

**Figure 1:**
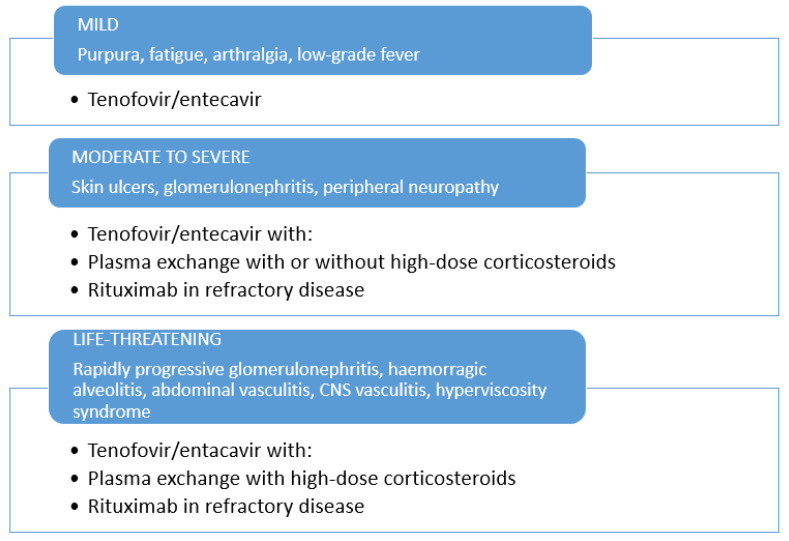
Therapeutic management of HBV-related cryoglobulinemic vasculitis.

NAs therapy should be preferred for the therapeutic management of HBV-related CV patients, in subjects with mild-to-moderate manifestations. The administration of short courses of low doses of corticosteroids may be considered in mild-to-moderate HBV-related CV (Figure 1), while long-term use of corticosteroids is discouraged. For severe cases, therapeutic measures applied in severe HCV-related or non-infectious CV may be borrowed with some differences (63). In severe cases, plasma exchange with high-dose corticosteroids should be combined with NAs. Rituximab should be considered as a subsequent option for non-responders to the antiviral therapy, or relapsing patients. To prevent dangerous HBV flare, rituximab administration always requires the association with NAs therapy (Figure 1). In life-threatening CV (i.e., rapid progressive glomerulonephritis, gastrointestinal vasculitis, acute hyper-viscosity syndrome) rituximab and/or plasma exchange with high-dose corticosteroids should be combined with NAs. Nonetheless, further investigations in a larger population on the combination of NAs with second-line therapy options will be valuable to set up the most appropriate strategy for the therapeutic management of HBV-related CV.

## Figures and Tables

**Table 1:** Summary of the clinical-serological and virological characteristics reported by the main studies on HBV-related CV.

	First Author, Year, Ref.		
	Boglione et al. 2015 [28]	Mazzaro et al. 2016 [29]	Li et al. (2017) [30]
**Number of Patients**	**7**	**17**	**12**
Female/male	¾	10/7	4/8
Age/years, median (range)	60 (49–65)	56 (45–70)	47(29–68)
**Clinical Features**			
Purpura, n (%)	3 (43)	17 (100)	7 (58)
Arthralgias, n (%)	0	12 (71)	3 (25)
Raynaud’s phenomenon, n (%)	0	3 (14)	0
Sicca Syndrome, n (%)	0	2 (9)	0
Skin Ulcers, n (%)	2 (29)	1 (6)	0
Peripheral neuropathy, n (%)	4 (57)	5 (29)	2 (17)
Glomerulonephritis, n (%)	0	3 (18)	12 (100)
Gastrointestinal vasculitis, n (%)	0	0	2 (17)
Chronic hepatitis, n (%)	NA	8 (47)	NA
Cirrhosis, n (%)	NA	5 (29)	NA
**Biochemical and Virological Features**			
MC type II/type III	NA	15/2	3/9
Cryocrit %, median (range)	3.4 (2.5–6)	3 (1–14)	NA
Rheumatoid Factor IU/mL, median (range)	NA	119 (88–5850)	694 (67–2730)
C4 mg/dl, median (range)	NA	8.0 (4–31)	6.0
ALT IU/mL, median (range)	79 (68–105)	71 (39–82)	44 (10–102)
Creatinine mg/dl, median (range)	NA	1.0 (0.7–1.2)	2.8 (0.0–9.8)
HBV-DNA positive, n (%)	7 (100)	17 (100)	12 (100)
HBsAg positive, n (%)	7 (100)	17 (100)	10 (83)

MC, mixed cryoglobulinemia; NA, data not available.

**Table 2:** Nucleotide analogues (NAs) therapy in patients with HBV-related cryoglobulinemic vasculitis.

Author, Year	Pts n.	Antiviral Agent, Dose Duration, Weeks (w), (n)	Other Treatment, (n)	Negative HBV-DNA	Laboratory Features	Clinical Manifestations, (n)	Immune Response/ ALT Response	Cryoglobulinemic Vasculitis Response,(n)
					Before Treatment		After Treatment	
Cakir et al. 2006 [35]	1	Lamivudine 100 mg/day = 76 w; Adefovir 10 mg/day = 108 w		100%	Cryocrit: Pos; RF:1110; C4:7; ALT: 125;	Purpura, Fatigue, Arthralgia, Cirrhosis	Cryocrit: Neg RF: normal ALT: normal	CR: Purpura; Fatigue Arthralgias;
Kawakami et al. 2008 [36]	1	Entecavir 0.5 mg/day		100%	Cryocrit: Pos	Purpura, Neuropathy,	Cryocrit: Neg	CR: purpura, Neuropathy
Enomoto et al. 2008 [37]	1	Entecavir 0.5 mg/day = 20 w		100%	Cryocrit: Pos	Purpura, Chronic hepatitis	Ccryocrit: Neg ALT: normal	CR: Purpura
Conca et al. 2009 [38]	1	Lamivudine 100mg/day = 4 w; Lamivudine 50 mg/day = 232 w		100%	Cryocrit: 7; RF: 876; C4:0.4; ALT:247	Purpura, Cirrhosis	Cryocrit: Neg ALT: normal	CR: Purpura
D’Amico et al. 2013 [39]	2	Tenofovir 245 mg/day = 200 w, (1); Entecavir 0.5 mg/day = 204 w, (1)		100%	Type III; Cryocrit: Pos; RF: Pos; C4:Pos	Purpura, (2); Neuropathy, (2); Chronic hepatitis,(2);	Cryocrit: Neg(2) RF: normal (2) C4: normal (2) ALT: normal (2)	CR: Purpura, (2); NR: Neuropathy, (2)
Boglione et al. 2013 [28]	7	Telbivudine 600 mg/day = 48 w, (7)		100%	Cryocrit: 3.4; ALT: 79	Purpura, (3); neuropathy, (4); Skin ulcer, (2); Chronic hepatitis, (7)	Cryocrit: 1% (0-2) ALT median: 33 (22–44)	CR: Purpura,(3); Neuropathy, (2); NR: Peripheral neuropathy, (2); Skin ulcer, (2)
Viganò et al. 2014 [40]	1	Entecavir 0.5 mg/72 h = 108 w		100%	Cryocrit: 3; RF: Pos; C4: 5; ALT: 178; creatinine: 3.4 mg/dl; proteinuria: 2.5 g/24 h	Purpura, Fatigue, GN, Cirrhosis	Cryocrit: Neg RF: normal C4: normal ALT: 13: Creatinine: 0.5 mg/dlproteinuria: 40 mg/day	CR: Purpura; Fatigue; GN
Yamazaki et al. 2014 [41]	1	Entecavir 0.5 mg/day = 28 w	CS+PE,	100%	Type II; Cryocrit: 2%; C4: 1; ALT: 4; creatinine: 4.0 mg/dl	Purpura, Skin ulcer, GN	Cryocrit: Neg	CR: Purpura, skin ulcers; NR: GN
Terrier et al. 2015 [22]	3	Lamivudine 100 mg/day, (1); Entecavir 0.5 mg/day, (2);	PE+CS+RTX, (1); PE+CYC+CS+RTX, (1)	100%2	Type II; Cryocrit: pos; C4: 0.24	Purpura, (2); Arthralgia, (2); GN, (3); Chronic hepatitis, (3)	Cryocrit: Neg (1)	CR: Purpura, (2); Arthralgia, (2); GN, (3);
Visentini et al. 2016 [42]	1	Tenofovir 245 mg/day = 52 w		100%	Type II; Cryocrit: pos; RF: pos; C4 low level	Purpura, Chronic hepatitis	Cryocrit: Neg RF: normal C4: low level	CR: Purpura
Mazzaro et al. 2016 [29]	7	Entecavir nr = 192 w, (5); Adefovir nr = 48 w, (1); Lamivudine = 192 w, (1)	CS alone previous NAs, (1)	100%	Type II, 7; Cryocrit: 3; RF: 200; C4: 8; ALT: 72	Purpura, 7; Arthralgia, 7; Skin ulcer, 1; Chronic hepatitis, 6; Cirrhosis, 1	Cryocrit median: 1% RF median: 86; C4 median: 10 ALT median: 20	CR: Purpura, (7); Arthralgia, (5); Skin Ulcer, (1); NR: Arthralgia, (2)
Li et al. 2017 [30]	9	Entecavir nr = 64 w, (7); Lamivudine nr = 24 w, (2)	CS alone, 3; CS+CYC, 1; CS+PE+RTX, (1); CS+PE+MMF, (1)	100%	Type II, 3; Type III, 6; Cryocrit: 1900 mg/L RF: 824; C4: 6; ALT: 48; Creatinine: 2.2 mg/dl; Proteinuria: 5.0 g/day	Purpura, (4); Arthralgia, (2); Neuropathy, 2; Gastrointestinal, 2; GN, 9;	Creatinine median: 1.0 mg/dl; Proteinuria median: 1.6 g/day.	CR: Purpura, (2); Arthralgia, (2); GN, (2); Neuropathy, (2); PR: GN, (3); Gastrointestinal, (1); NR: GN, (4); Gastrointestinal, (1);

Legend: RF, rheumatoid factor, normal range: 0–25 IU/mL; C4, complement fraction C4, normal range: 10–40 mg/dl; ALT, alanine aminotransferase, normal range: 6–36 IU/L; GN, Glomerulonephritis, CS, Corticosteroids, CYC, cyclophosphamide, RTX, Rituximab, MMF, mycophenolate mofetil, PE, plasma exchange.

**Table 3:** Nucleotide analogues (NAs) therapy in 18 patients with HBV-related cryoglobulinemic vasculitis.

**No. of Patients**	**18**
**Female/Male**	10/8
**Age/Years, Median (range)**	59 (33–81)
**Biochemical and Virological Features**	
HBV-DNA positive, n (%)	18 (100)
HBV-DNA IU/mL, median	6630
MC type II/type III	17/1
Cryocrit %, median (range)	4 (1–70)
RF IU/mL, median (range)	181 (10–5850)
C4 mg/dl, median (range)	9 (2–31)
ALT IU/mL, median (range)	51 (21–638)
Creatinine mg/dl, median (range)	1.0 (0.6–1.3)
**Clinical Features**	
Purpura, n (%)	18 (100)
Arthralgias, n (%)	11 (61)
Skin Ulcers, n (%)	3 (17)
Sjögren’s syndrome, n (%)	5 (28)
Peripheral neuropathy, n (%)	11 (61)
Glomerulonephritis, n (%)	1 (6)
NHL	2 (11)
Chronic hepatitis, n (%)	4 (22)
Cirrhosis, n (%)	1 (6)
**Antiviral Agent, n, Median Duration (months)**	
Entecavir	11 (78)
Tenofovir	6 (67)
Lamivudine	1 (58)
**Other treatment, n, Median Duration (months)**	
Peg-IFN alone	3 (17)
CS-associated NAs	4 (22)
PE-associated NAs	4 (22)
RTX-associated NAs	2 (11)
**Virological Response, n (%)**	18 (100)
Cryocrit %, median (range)	1 (0–14)
RF IU/mL, median (range)	181 (10–5850)
C4 mg/dl, median (range)	7 (1–24)
ALT IU/mL, median (range)	16 (12–34)
**Cryoglobulinemic Vasculitis Complete Response**	
Purpura, n (%)	14 (78)
Arthralgia, n (%)	8 (44)
Skin Ulcers, n (%)	2 (11)
Sjögren’ssyndrome, n (%)	2 (11)
Peripheral neuropathy, n (%)	6 (33)
**Cryoglobulinemic Vasculitis Partial Response**	
Purpura, n (%)	4 (22)
Arthralgia, n (%)	3 (17)
Peripheral neuropathy, n (%)	5 (28)
Glomerulonephritis, n (%)	1 (6)
NHL	2 (11)

Legend: CS, Corticosteroids, CYC, cyclophosphamide, RTX, Rituximab, PE, plasma exchange, NHL, non-Hodgkin lymphoma, RF, rheumatoid factor. Normal range: RF (0–25 IU/mL); C4 (10–40 mg/dl); ALT (6–36 IU/L).

**Table 4:** Clinical features and pathologic counterpart in cryoglobulinemic vasculitis.

	Clinical Features	Most Common Pathologic Features
Mild disease	Lower limb purpura	Small vessel leucocytoclastic vasculitis
	Fatigue	
	Arthralgia	
	Low-grade fever	
Moderate-to-severe disease	Widespread purpura	
	Sensory–motor peripheral neuropathy	
	Mononeuritis	Vasa nervorum vasculitis
	Renal failure	Type I membranoproliferative glomerulonephritis
	Skin ulcers	Small vessel necrotizing vasculitis
Life-threatening disease	Alveolar haemorrhage	Pulmonary capillaritis
	Abdominal vasculitis	
	Rapid progressive renal failure	Type I membranoproliferative glomerulonephritis with widespread crescentic extracapillary proliferation
	Central nervous system vasculitis	
	Hyperviscosity syndrome	

## Data Availability

All data available are published. Further details can be directly asked from the Corresponding Author.
